# Generation of myostatin edited horse embryos using CRISPR/Cas9 technology and somatic cell nuclear transfer

**DOI:** 10.1038/s41598-020-72040-4

**Published:** 2020-09-24

**Authors:** Lucia Natalia Moro, Diego Luis Viale, Juan Ignacio Bastón, Victoria Arnold, Mariana Suvá, Elisabet Wiedenmann, Martín Olguín, Santiago Miriuka, Gabriel Vichera

**Affiliations:** 1 LIAN-CONICET, Fundación FLENI, Buenos Aires, Argentina; 2 Consejo Nacional de Investigaciones Científicas y Técnicas (CONICET), Buenos Aires, Argentina; 3 Laboratorio de Neurología y Citogenética Molecular, CESyMA, Buenos Aires, Argentina; 4 KHEIRON BIOTECH S.A, Pilar, Buenos Aires, Argentina

**Keywords:** Biotechnology, Cell biology, Developmental biology, Genetics, Molecular biology

## Abstract

The application of new technologies for gene editing in horses may allow the generation of improved sportive individuals. Here, we aimed to knock out the myostatin gene (*MSTN*), a negative regulator of muscle mass development, using CRISPR/Cas9 and to generate edited embryos for the first time in horses. We nucleofected horse fetal fibroblasts with 1, 2 or 5 µg of 2 different gRNA/Cas9 plasmids targeting the first exon of *MSTN*. We observed that increasing plasmid concentrations improved mutation efficiency. The average efficiency was 63.6% for gRNA1 (14/22 edited clonal cell lines) and 96.2% for gRNA2 (25/26 edited clonal cell lines). Three clonal cell lines were chosen for embryo generation by somatic cell nuclear transfer: one with a monoallelic edition, one with biallelic heterozygous editions and one with a biallelic homozygous edition, which rendered edited blastocysts in each case. Both *MSTN* editions and off-targets were analyzed in the embryos. In conclusion, CRISPR/Cas9 proved an efficient method to edit the horse genome in a dose dependent manner with high specificity. Adapting this technology sport advantageous alleles could be generated, and a precision breeding program could be developed.

## Introduction

In the last 17 years, horse cloning has focused on multiplying valuable individuals mainly because of commercial interests^1^, Kheiron S.A (www.kheiron-biotech.com), ViaGen (www.viagen.com)]. The strongest advantage of this technique is the genome conservative property, so that the cloned foals invariably “inherit” the original genotype. However, this technique can be even more powerful when it is combined with gene editing, to obtain horses with the genetic background of the original individual and new desired characteristics in one generation and in a non-random way.

The clustered regularly interspaced short palindromic repeats (CRISPR) system is one of the techniques for gene editing that has successfully been used to improve animal features and to obtain desired genotypes in different species. This system was first described in prokaryotes as an acquired immune system against plasmids and phages^2,3^. Once discovered, this natural system was adapted to become a tool for gene editing in kingdoms as distant as fungi, plants and animals^4–6^. By this technique, numerous genome edited animals from different species have been generated^7^. These animals were modified to be potentially immune to infective diseases^8^, to serve as bioreactors^9^, for human disease modeling^10,11^ and for xenotransplantation^12,13^.

The main interest in genetically modified horses focuses on disease resistance, genetic disease reversion and sportive performance improvement. Recently, a deleterious mutation in the *GBE 1* gene that causes an autosomal recessive condition known as glycogen branching enzyme deficiency and a single nucleotide mutation in the *PPIB* gene that causes a skin disease in horses were edited in horse fibroblasts by homologous recombination with CRISPR/Cas9^14,15^. In both cases, the normal genotype was restored with a view to later using these cells for cloning. However, no reports on CRISPR/Cas9 application to sportive performance are available so far and targeting the myostatin gene (*MSTN*) constitutes one of the most promising approaches.

**Figure 1:**
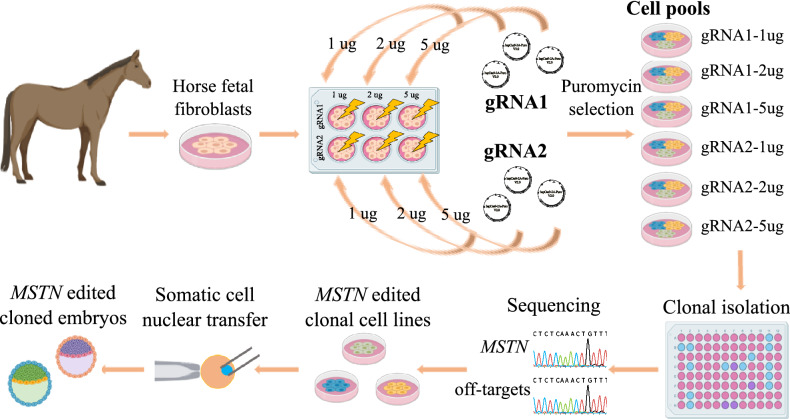
Experimental design for the generation of *MSTN*-KO cloned embryos in the horse. Horse fetal fibroblasts were nucleofected with different concentrations of the CRISPR/Cas9 system containing two gRNAs (gRNA1 and gRNA2) directed to the first exon of the myostatin gene (*MSTN*). After puromycin selection, the surviving cells (cell pools) for each condition were subjected to clonal culture and subsequently sequenced for *MSTN*. Three *MSTN* edited clonal cell lines were chosen for embryo generation by somatic cell nuclear transfer (SCNT): one clonal cell line with a monoallelic edition, one with biallelic heterozygous editions and one with a biallelic homozygous edition. *MSTN* edited blastocysts were obtained.

MSTN is a negative regulator of muscle growth and differentiation^16^. It is expressed in skeletal muscle^17^ and mutations in its sequence result in augmented muscle mass. Natural *MSTN* gene mutations with this phenotype are present in cattle breeds such as the Belgian Blue and Piedmontese^18^, and in dogs^19^. This gene has also been experimentally edited using CRISPR in different mammals including pigs^20,21^, dogs^22^, rabbits^23^, goats^24,25^ and sheep^26^, and using TALEN in cattle and sheep^27^. Most of these studies used zygote microinjection to generate the *MSTN*-knock out (*MSTN*-KO) genotype. However, this approach may produce mosaic embryos, which may make it difficult to anticipate the mutations generated and/or putative off-targets (OTs) before animal birth, as it would require embryo biopsy followed by whole genome amplification to analyze the genotype of the edited embryo. For these reasons, cloning is postulated as a promising technique to generate gene edited animals as the cell line with the desired edition can be selected before the somatic cell nuclear transfer (SCNT) procedure. This technique becomes even more relevant in species such as the horse, in which embryo generation by intracytoplasmic sperm injection (ICSI) is inefficient^28,29^, and the successful of embryo generation by in vitro fertilization (IVF) is not reliable^30,31^.In this work we aimed to generate edited horse embryos by knocking out *MSTN* using CRISPR/Cas9 and SCNT (Fig. 1). To our knowledge, this is the first time that edited horse embryos have been generated. This strategy allowed us to choose those clonal cell lines with mono- or bi-allelic editions and no off-targets to be used as nuclear donors for cloning.

## Results

### Horse fetal fibroblasts nucleofection efficiency

In order to estimate the nucleofection efficiency of horse fetal fibroblasts (HFFs) using the NEON system, we compared different concentrations of the EGFP-N1 plasmid. With this experiment, EGFP expression above 87% was observed by flow cytometry in all conditions (Supplementary Figure S1).

### Edition efficiency was dependent on the gRNA and the plasmid concentration

**Figure 2:**
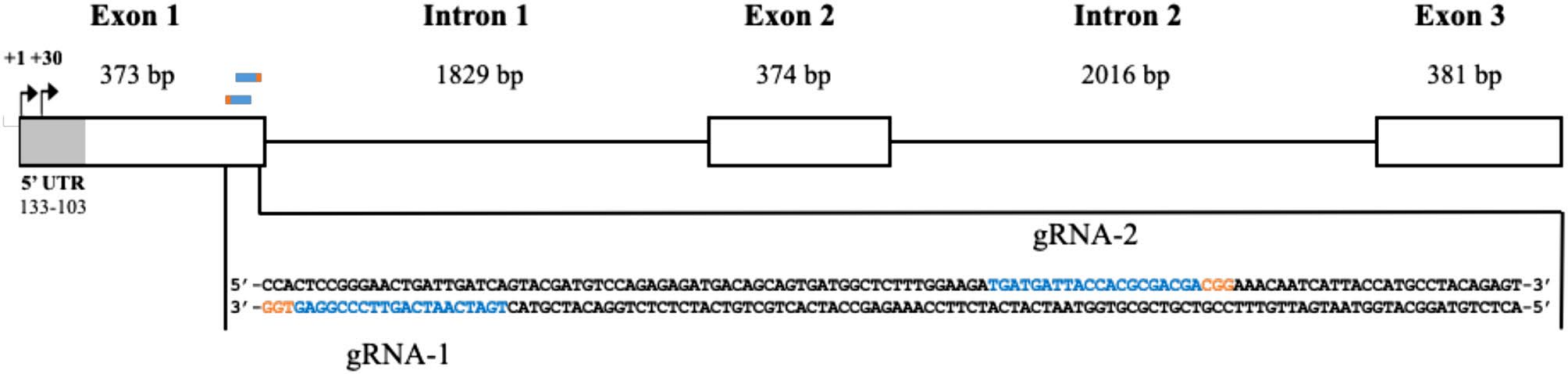
Schematic representation of horse *MSTN* gene and gRNAs design. The white boxes and lines represent exons and introns, respectively. Inserted grey box represents 5′ untranslated region (5′UTR). The sequence below represents part of exon 1 containing Cas9/gRNA target sites for gRNA1 and gRNA2 labeled in blue. Protospacer-adjacent motif (PAM) is labeled in orange. Black arrows represent transcription start sites.

**Figure 3:**
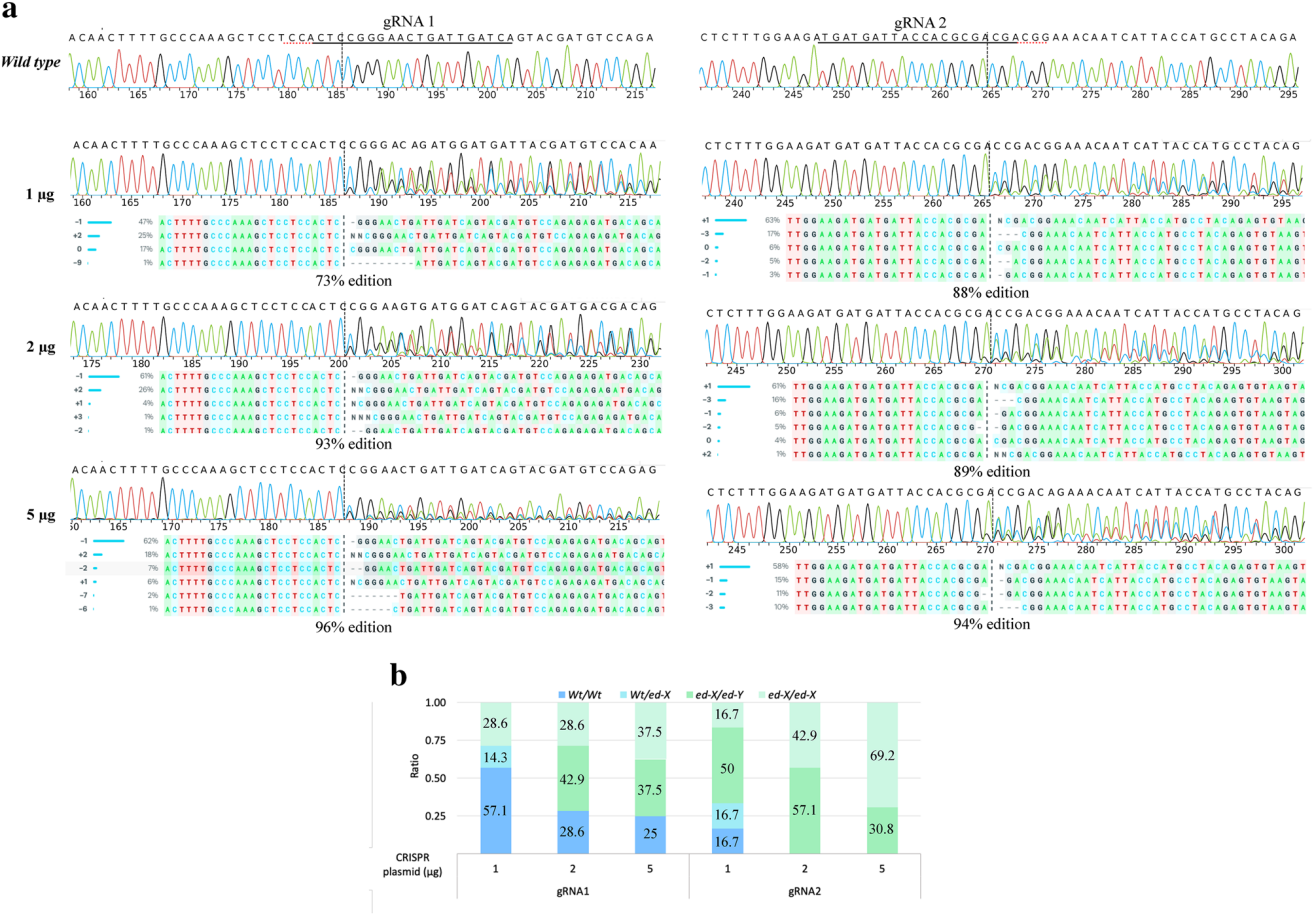
*MSTN* gene editions with gRNA1 and gRNA2. (**a**) Histograms with nucleotide sequence data of cell pools edited with 1, 2 and 5 µg per 1 × 10^6^ cells of CRISPR system. Different edited genotypes predicted by InDels analysis (ICE, Synthego) and percentage of edition for each condition. (**b**) Ratio of *MSTN* genotype in cell clones with both gRNAs and different plasmid concentrations. *Wt/wt*, cell clones with no editions; *Wt*/*ed-X*, cell clones with monoallelic editions; *ed-X/ed-Y*, cell clones with biallelic heterozygous editions; *ed-X/ed-X* cell clones with biallelic homozygous editions.

Two gRNAs were designed to target exon 1 of equine *MSTN* (Fig. 2). The edition efficiency of each gRNA was first evaluated in HFF puromycin resistant cells (cell pools) by PCR amplification (Table 1) of the target region followed by Sanger sequencing. Both gRNAs were able to generate editions in the first exon of *MSTN*. Moreover, different concentrations of the plasmid were used for nucleofection. According to insertions and deletions (InDel) analysis by Synthego`s Inference of CRISPR Edits (ICE) tool^32^, edition efficiencies were 73%, 93% and 96% for gRNA1 and 88%, 89% and 94% for gRNA2 in cell pools when 1, 2 and 5 µg per 1 × 10^6^ cells were used, respectively (Fig. 3A). Therefore, increasing plasmid concentration improved edition efficiency. To further characterize both gRNAs, InDel characteristics were evaluated in the isolated clonal cell lines. We obtained different genotypes depending on the gRNA and the plasmid concentration used (Fig. 3B and Supplementary Table S1). The best condition for *MSTN* double allele-KO was obtained with gRNA 2 using 5 µg plasmid (11/13 cell clones, not considering alleles containing triplet InDels as KO). Moreover, large insertions corresponding to the nucleofected plasmid sequence were observed in 4 clonal cell lines: G1-2µg-C06, G1-5µg-C02, G2-5µg-C05 and G2-5µg-C18; and large insertions corresponding to chr15 and chr29 genomic regions were observed in 2 clonal cell lines: G2-2µg-C29 and G2-2µg-C28, respectively.

**Table 1:** Primer sequences for myostatin gene (*MSTN*) and putative off-targets (OT1 and OT2) for gRNA1 and gRNA2.

Target	Primer Sequences	PCR fragment
*MSTN*	F: 5′-TTGTGCTGATTCTTGCTGGTC-3′	654 bp
	R: 5′-CCCAATTTTTGCCTTGGTGGT-3′	
*OT1 gRNA1*	F: 5′-CCAATGCCACATTCAACGA-3′	714 bp
	R: 5′-AGAGGGGGTTAAGGGCTGTT-3′	
*OT2 gRNA1*	F: 5′-GGGAGGAATTGAGCCACGAA-3′	483 bp
	R: 5′-TTCAGTTCCTCTTGACGGGC-3′	
*OT1 gRNA2*	F: 5′-ACCAGCTTTGACTTAGAAAATCC-3′	432 bp
	R: 5′-ATTTGGTTCAAAGATGCTCCTGT-3′	
*OT2 gRNA2*	F: 5′-ACATGGATTTTAGAGCCAGTCAGA-3′	400 bp
	R: 5′-AGGTCACTATTAGAAAGGCAAATTC-3′

The genotypes obtained are summarized in Fig. 3B. Overall, clonal cell lines characterization resulted in an average efficiency of 63.7% (14/22 edited clonal cell lines) for gRNA1 and 96.1% (25/26 edited clonal cell lines) for gRNA2 considering the three experimental conditions.

### Putative off-targets evaluation in cell pools and clonal cell lines

**Figure 4:**
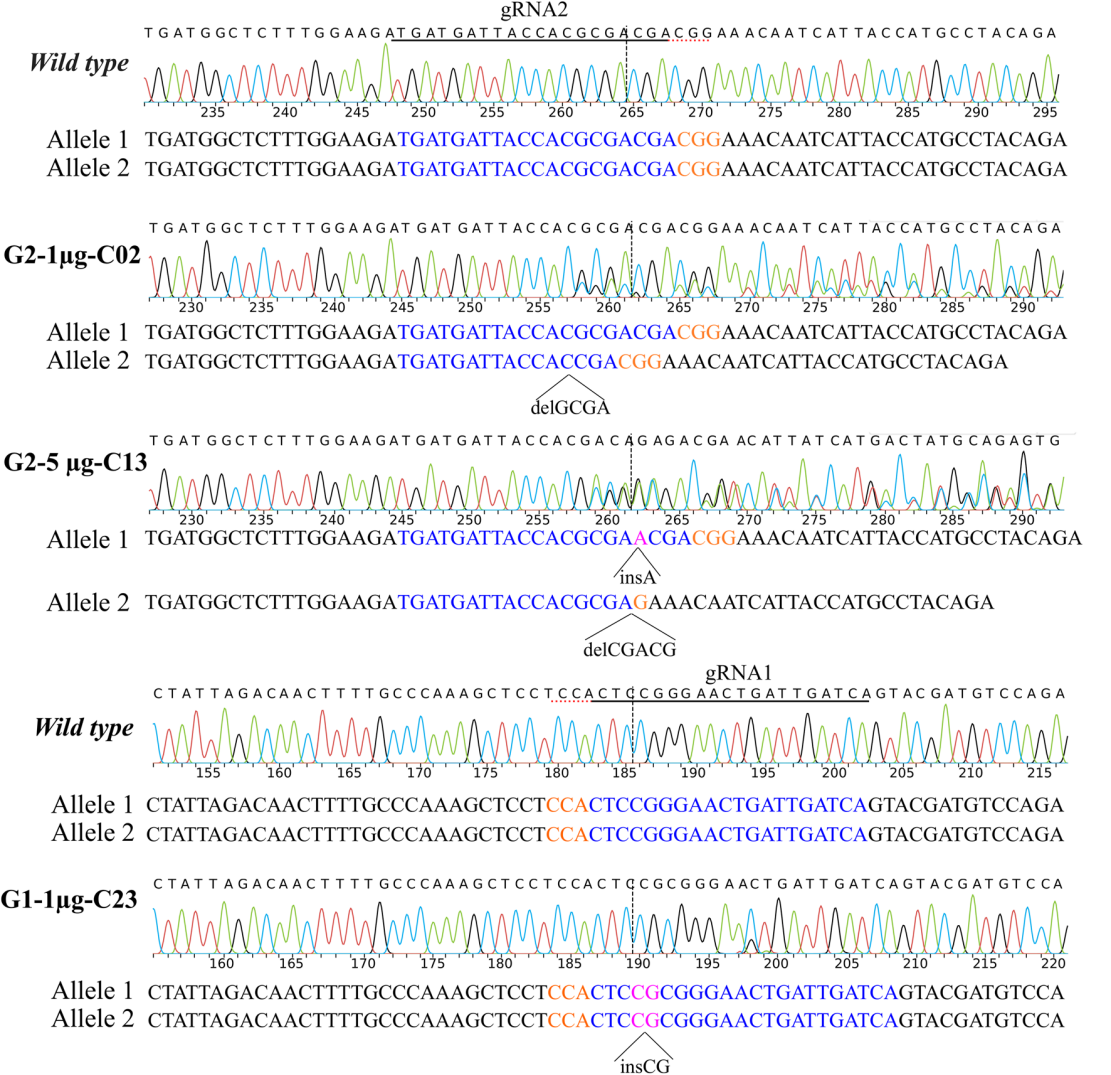
*MSTN* edited sequences of three cell lines chosen for embryo generation by SCNT. Sanger sequencing of three cell lines with different editions. gRNA1 and gRNA2 sequences are shown in blue with the dotted line pointing the cut site of Cas9 for each gRNA. The protospacer-adjacent motif (PAM) is labeled in orange. Both deletions (*del*) or insertions (*ins*) are detailed for each cell line.

**Figure 5:**
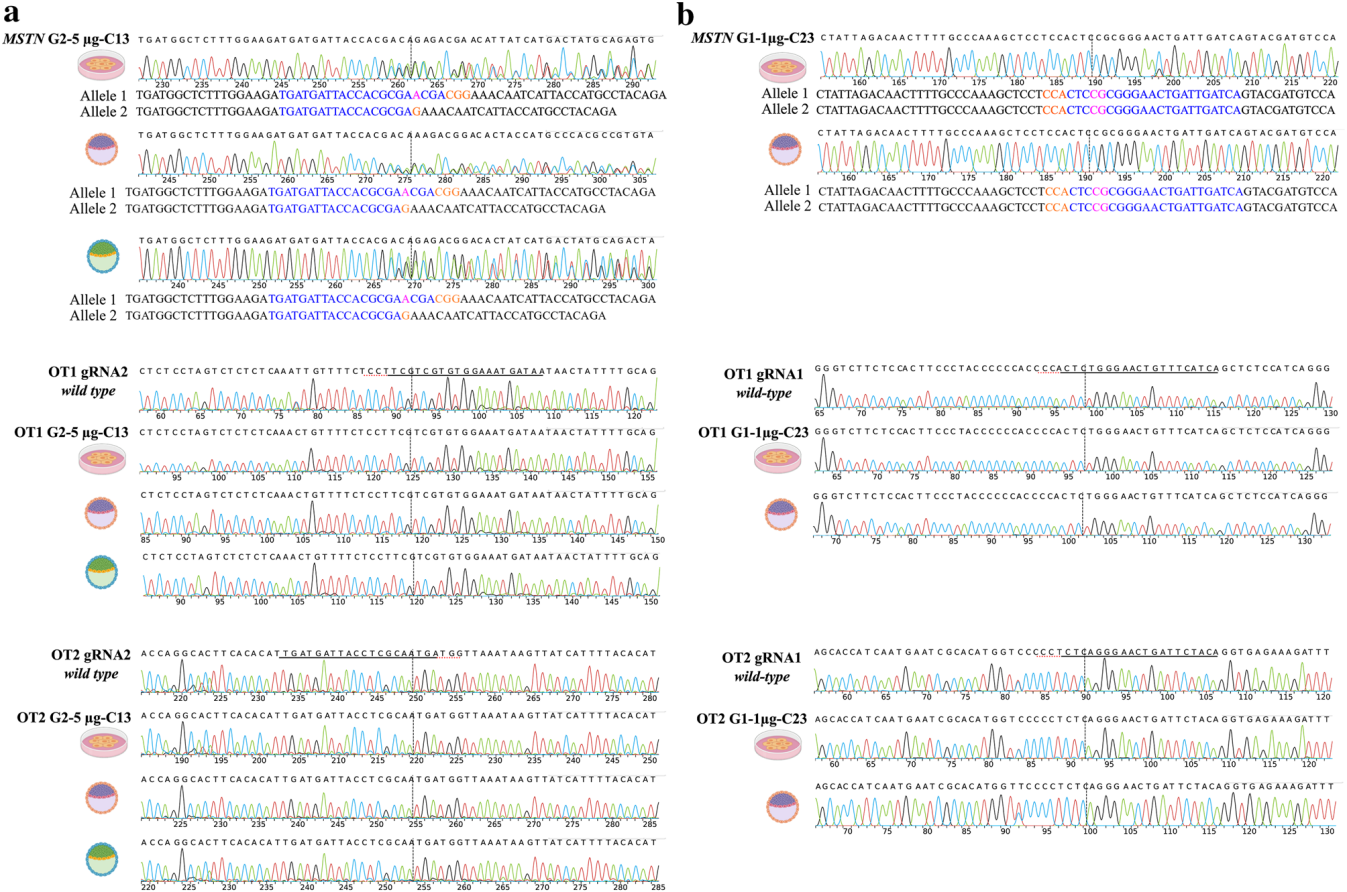
*MSTN* and two putative off-target sequences in edited clonal cell lines and blastocysts generated by SCNT. (**a**) Chromatograms obtained by Sanger sequencing of G2-5 µg-C13 *MSTN* edition and two putative off-targets (OT1 and OT2) in the clonal cell line and two blastocysts obtained after SCNT with these cells as nuclear donors. (**b**) Same analysis as in (**a**) in the G1-1 µg-C23 group, in this case on only one embryo.

One of the advantages of edited embryo generation by SCNT is the possibility of characterizing the donor cell lines for the gene edition and the absence of OTs. First, we analyzed two high rank putative off-targets (OTs) [according to Benchling online software (https://www.benchling.com)] of each gRNA in the six experimental cell pools. After InDel analysis, OTs were only observed in the OT1 of gRNA1-5 µg cell pool, but not in the other 5 experimental groups (Supplementary Figure S2). As OTs accounting for less than 5% may go under detected in a cell pool by Sanger sequencing, the edited clonal cell lines chosen for embryo generation (described below) (Fig. 4) were also subjected to OTs evaluation, revealing no differences with respect to the *wild-type* control (Fig. 5).

### *MSTN*-KO embryo generation by SCNT

**Figure 6:**
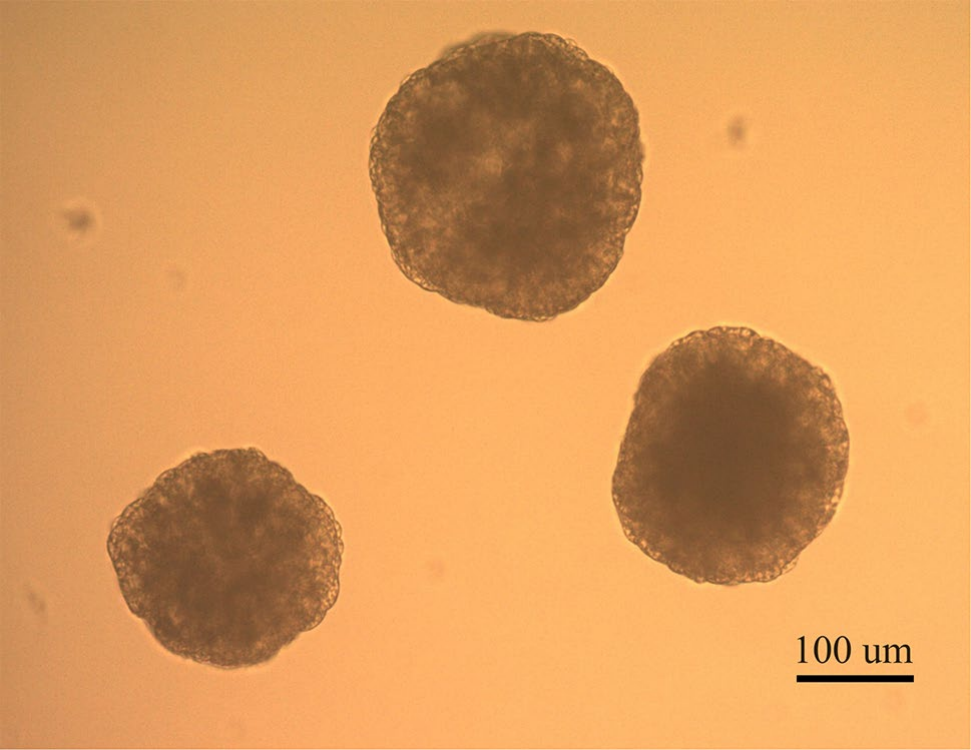
*MSTN* knock-out horse embryos. Three day 7 horse embryos obtained from G1-1 µg-C23 experimental group.

To evaluate the developmental capacity of the embryos produced using edited cells, three clonal cell lines were selected for further characterization and embryo generation by SCNT. The chosen clonal cell lines were G2-1 µg-C02 (with a monoallelic edition), G2-5 µg-C13 (with biallelic heterozygous editions) and G1-1 µg-C23 (with a biallelic homozygous edition). The edited *MSTN* sequences of each clonal cell line are detailed in Fig. 4, whereas the results of embryo development are summarized in Table 2. The three clonal cell lines were able to generate blastocysts (Fig. 6), although with lower efficiency than mesenchymal stem cells (MSCs) (*p* < 0.05) and a non-statistically significant tendency to lower efficiency than the *wild-type* HFF control. In addition, three of the blastocysts generated were evaluated for *MSTN* edition and putative OTs, with results showing the same *MSTN* sequence in the embryos and the clonal cell lines, without OTs (Fig. 5).

**Table 2:** Equine blastocysts produced by SCNT of CRISPR/Cas9 edited cell clones.

Cell line*	n	Cleavage (%)	Blastocysts (%)
G2-1 µg-C02 (*Wt*/ed-X)	153	102 (67)^a^	3 (2)^a^
G2-5 µg-C13 (ed-X/ed-Y)	155	108 (70)^ba^	3 (1.9)^a^
G1-1 µg-C23 (ed-X/ed-X)	159	133 (84)^c^	3 (1.9)^a^
Fibroblasts (*Wt*/*Wt*)	140	120 (86)^c^	8 (5.7)^ab^
MSCs (*Wt/Wt*)	73	59 (81)^bc^	9 (12.3)^b^

*Wt/ed-X, cell line with a monoallelic edition; ed-X/ed-Y, cell line with biallelic heterozygous editions; ed-X/ed-X cell line with a biallelic homozygous edition, *Wt/wt*, no editions. MSCs, mesenchymal stem cells.

^a,b,c^Values with different superscripts in a column are significantly different (Fisher’s exact test *p* < 0.05).

## Discussion

*MSTN* has been studied and edited in different mammalians’ species mainly with the purpose of increasing meat production in cattle^27^, goats^24,25^, sheep^26,27^ and pigs^20,21^, and for enhancing sport performance in dogs^22^. In addition, some cattle and dog breeds have natural MSTN loss of function mutations^18,19^. In horses, such mutations have been neither described nor generated, although one single nucleotide polymorphism (SNP) in the *MSTN* intron sequence has been identified^33^. This SNP has been associated with fitness at different racing distance ranges and with muscle fiber proportions^33–36^. However, it was later demonstrated that this SNP was linked to a short interspersed nuclear element insertion (SINE insertion) in the *MSTN* gene promoter^36,37^, which alters the transcription start site and, consequently, the transcript levels of the gene^37^. It has been shown that *MSTN* expression levels affects the performance ability of each individual, giving faster horses for shorter distances with lower *MSTN* expression^38^. On the basis of these studies, we decided to edit *MSTN* to generate KO horse embryos by SCNT and consider other point editions in the future that could enhance sport performance in horses.

First, we evaluated two gRNAs targeting the first exon of *MSTN*. We observed high nucleofection efficiency in HFFs with the EGFP-N1 plasmid using the Neon system and we determined that both gRNAs were suitable for generating InDels in the gene, albeit with different efficiencies. According to InDels analysis (ICE, Synthego)^32^ of the cell pools and considering the three different concentrations evaluated, the average efficiencies were 87.33% for gRNA 1 and 90.33% for gRNA2. In addition to gRNA efficiencies, the three experimental concentrations of the plasmid displayed different capabilities to generate InDels in exon1 of *MSTN*. Both in cell pools and in the individual analysis of clonal cell lines, more editions were observed as the concentration of the plasmid increased. However, high plasmid concentration induced undesired OTs in G1-5 µg conditions and insertions corresponding to plasmid DNA delivered-edition in four clonal cell lines. These kind of insertions were previously described in a hornless genome-edited bull generated by TALEN and a plasmid HDR-donor sequence after whole genome sequencing analysis^39,40^. In addition, monoallelic editions were obtained only when the lowest plasmid concentration was used (1 µg per 1 × 10^6^ cells), and higher proportion of biallelic editions were identified in clonal cell lines with higher concentrations of the CRISPR system. Similar results have been reported in pig embryos when different concentrations of Cas9 protein and gRNA were used for zygote microinjection^41^. These results strongly suggest that the concentration of CRISPR/Cas9 plasmid or ribonucleoprotein complex directly affects gene editing efficiency and it could be used as a methodological strategy to generate mono- or biallelic editions.

Reproductive biotechnology applied to the generation of edited embryos also affects the overall efficiency. Until now, no reliable protocols have been made available for IVF in horses and an efficient method to edit embryos by ICSI has not been yet developed. Moreover, zygote microinjection has proven to have high rates of mosaic embryos/animals^8,42–46^, OT occurrence^26,47^ and low birth rates of edited animals^48,49^. In sheep, for example, the reported efficiencies to disrupt the *MSTN* gene using CRISPR/Cas9 by zygote microinjection were 5.7%^48^, 45.4%^26^ and 27.7%^49^. Therefore, we chose the cloning technique as it allows the analysis of the edited gene sequence and putative OT activity prior to the generation of the embryos^50,51^. In this work, we confirmed the absence of two high ranked putative OTs in five of the six cell pools and in the clonal cell lines used to generate the edited embryos.

Despite the great advantages of cloning, one of its disadvantages is the need to generate clonal cell lines, which requires increasing cell passages during the isolation and expansion process. In horses, the rate of nuclear remodeling decreases significantly after embryo reconstruction using fetal fibroblasts of increased passage number^52^. Then, in addition to the low blastocyst rates reported for horse cloning, this factor might explain the lower embryo developmental rate of the edited cloned embryos compared to controls. To increase horse cloning development, MSCs could be used as nuclear donors^1^. However, it was demonstrated that MSCs undergo senescence at early passages, showing alterations in cellular morphology, telomere shortening and proliferation arrest after 30 population doublings or 7–10 passages^53^, which makes the generation of edited clonal cell lines rather difficult.

In summary, we demonstrate that it is possible to edit HFFs by CRISPR/Cas9 with high efficiency and generate embryos with genetic modifications at the blastocyst stage by SCNT. To our knowledge, edited horse embryos had not been reported until now. With this technique available other editions could be achieved, including the correction of genetic defects that cause equine diseases^15,54^. Our long-term goal is then to identify natural sport-advantageous allele sequences present in the genome of some individuals and incorporate them in others to endow them with the desired characteristics. In this way, we could introduce the SINE insertion in the *MSTN* promoter of those animals lacking it, in order to alter the proportion of muscle fibers and obtain faster horses for short distances. We consider this a precision breeding strategy which can be achieved in only one generation.

## Methods

### gRNAs design and construction

Two gRNAs complementary to the first exon of the equine *MSTN* gene were designed using Benchling 2018 (https://www.benchling.com) (Fig. 2). The sequences were gRNA1: TGATCAATCAGTTCCCGGAG (chr18:66609845-66609864, EquCab3.0) and gRNA2: TGATGATTACCACGCGACGA (chr18:66609780-66609799, EquCab3.0). To obtain synthetic oligonucleotides codifying each gRNA, two complementary oligo DNAs were synthesized, annealed and cloned as previously described^55^. The backbone used was hspCas9-2A-Puro V2.0 plasmid, which consisted in U6-sgRNA and Cas9 expression elements. hspCas9-2A-Puro V2.0 was a gift from Feng Zhang (Addgene plasmid #62,988 https://www.n2t.net/addgene:62988; RRID: Addgene_62988). The constructs containing each gRNAs were confirmed by Sanger sequencing (Macrogen Inc., Korea).

### Cell culture and plasmid nucleofection

HFFs and MSCs were cultured in Dulbecco’s modified Eagle’s medium (DMEM, #11,885, Gibco, Grand Island, NY, USA) supplemented with 10% heat-inactivated fetal bovine serum (FBS, #16,000–044 Gibco, Grand Island, NY, USA). For each nucleofection procedure with HFFs, cells were allowed to grow until 80% confluence. First, cells were nucleofected with 1, 2 or 5 µg of EGFP-N1 plasmid (Clontech Laboratories, Mountain View, CA, USA) and co-nucleofected with each gRNA plasmid, in order to determine the nucleofection efficiency with this plasmid. Briefly, 1 × 10^6^ cells were nucleofected using the NEON Transfection system (Thermo Fisher Scientific, Massachusetts, USA) with a 100 µl tip on 3 pulses of 1,650 V, 20 ms. Forty-eight hours after nucleofection, the cells were trypsinized and analyzed by flow cytometry in a BD Accuri cytometer (excitation laser 488 nm and 533/30 filter). Once nucleofection success and efficiency had been confirmed, either gRNA1 or gRNA2 was nucleofected using 1, 2 or 5 µg of the plasmid (one experimental replicate). The experimental groups were: a) gRNA1-1µg, b) gRNA1-2µg, c) gRNA1-5µg, d) gRNA2-1µg, e) gRNA2-2µg and f) gRNA2-5µg. After 48 h, cells were treated with 2.5 µg/ml puromycin (#ant-pr 1, InvivoGene, CA, USA) for a 48-h period to select those cells that incorporated the plasmid (cell pools). Once puromycin-selected, the cell pools were clonally cultured by plating individual cells in 96 MW plates in DMEM medium supplemented with 10% FBS, 10 ng/ml bFGF (#RP-8627, Invitrogen, CA, USA) and 10 ng/ml IGF1 (#RP-10931, Invitrogen, CA, USA).

### Genomic DNA extraction and analysis of editions

Cell pools, clonal cell lines and putative edited blastocysts were subjected to DNA extraction by overnight lysis buffer incubation (10 mM Tris–HCl, 50 mM KCl, 2 mM MgCl2, 0,001% gelatin, 0.5% NP-40 and 0.5% Tween). Then, lysed cells and embryos were treated with 0.05 mg/ml Proteinase K (#25,530,049, Invitrogen, CA, USA) for 1 h at 37ºC and 15 min at 95ºC. The lysates were used for PCR analysis to determine *MSTN* editions and two putative OTs for each gRNA (OT1 and OT2). The primers sequences are listed in Table 1. *MSTN* primers were designed so that a 654 bp band was obtained with the gRNA1 edition site at 225 bp from the forward primer and the gRNA2 edition site at 303 bp from the forward primer. OT loci were selected according to the OT ranking of Benchling online software (https://www.benchling.com). OT specifications are summarized in Table 3. Once the DNA band was confirmed, the PCR product was used for reamplification by PCR, ethanol precipitation, isopropyl alcohol extraction and Sanger sequencing under standard protocol (service provided by Macrogen Inc., Korea). The sequences of PCR fragments were analyzed by TIDE (https://www.tide.nki.nl)^56^ and Synthego's ICE (https://www.ice.synthego.com)^32^ online software tools to evaluate InDels in each region compared to control cells. In clonal cell lines, these online tools could only analyze InDels up to 30–50 bp, respectively. For large InDels, sequence chromatograms were analyzed by Indigo online tool using *wild type* samples chromatograms as reference sequences (https://www.gear-genomics.com/indigo/)^57^. Analysis conditions were 50 base chromatogram trim size on each side and peak percentage to base call on 20%.

**Table 3:** gRNA1 and gRNA2 putative off-targets (OT1 and OT2) sequences and genomic position.

Off-targets	Sequences*	Mismatches	PAM	Loci
OT1 gRNA1	TGAT***G***AA***A***CAGTTCCC***A***GAG	3	TGG	chr1:-96298062
OT2 gRNA1	TG***TAG***AATCAGTTCCC***T***GAG	4	AGG	chr11:-47613259
OT1 gRNA2	T***T***AT***C***ATT***T***CCAC***A***CGACGA	4	AGG	chr1:-101621688
OT2 gRNA2	TGATGATTACC***T***CGC***A***A***T***GA	3	TGG	chr15:-84545182

* Mismatches are highlighted in bold italic.

### Generation of *MSTN* edited embryos by SCNT

Three edited cell lines were selected as nuclear donors for horse cloning. Two of them were *MSTN*-KO lines, one with the same edition in each allele (clone G1-1 µg-C23) and the other one with different editions in each allele (clone G2-5 µg-C13). The third cell line used was a heterozygous cell line with one edited allele (clone G2-1 µg-C02) (Fig. 4). Embryo generation by zona free nuclear transfer was performed as previously described by our group^58^. Briefly, ovaries were obtained from local slaughterhouses (Raul Aimar S.A., Ruta 36 km. 597, Río Cuarto, Córdoba, Argentina, ZIP code: 5805) and oocytes were matured for 24 h. Matured oocytes were then treated with pronase (#P-8811, Sigma Aldrich Co., USA) to remove the *zona pellucida*, enucleated by micromanipulation and electrically fused with a donor cell (*wild type* fibroblast, G1-1 µg-C23, G2-5 µg-C13 or G2-1 µg-C02 cell). In order to incorporate a positive control, we performed the same procedure using MSCs (with the same genomic background as the HFFs) as nuclear donors, considering the enhanced effectiveness of using this type of cells in horse cloning^1^. After 2.5 h fusion, reconstructed embryos were activated with 8.7 μM ionomycin (#I24222; Invitrogen, CA, USA) for 4 min followed by individual culture in a combination of 1 mM 6-dimethylaminopurine (6-DMAP; # D2629, Sigma Aldrich Co., MO, USA) and 5 mg/ml cycloheximide (CHX; #C7698, Sigma Aldrich Co., MO, USA) in 5 µl drops of DMEM/F12 (#D8062, Gibco, Grand Island, NY, USA) for 4 h. Reconstructed embryos were cultured in DMEM/F12 containing 10% FBS, and 1% penicillin–streptomycin in the Well-of-the-Well system, three embryos together per well. Two experimental replicates were performed with the *MSTN* edited cells (G1-1 µg-C23, G2-5 µg-C13 or G2-1 µg-C02) together with the *wild type* HFF control group in each procedure, and one replicate was performed comparing SCNT efficiency with MSCs and HFF wild type donor cells. Finally, embryo development was assessed on day 2 (cleavage rates) and on day 7 (blastocyst rates).

## Supplementary information

Supplementary information.
