# Supplementary material for: Generation of myostatin edited horse embryos using CRISPR/Cas9 technology and somatic cell nuclear transfer
Source: Sci Rep. 2020 Sep 24;10:15587. doi: 10.1038/s41598-020-72040-4 (PMC7518276; doi:10.1038/s41598-020-72040-4)
Supplement: Supplementary file 1 — Supplementary information. [file 41598_2020_72040_MOESM1_ESM.pdf]

Generation of Myostatin Edited Horse Embryos Using CRISPR/Cas9 Technology and Somatic Cell Nuclear Transfer

Lucia Natalia Moro, Diego Luis Viale, Juan Ignacio Bastón, Victoria Arnold, Mariana Suvá, Elisabet Wiedenmann, Martín Olguín, Santiago Miriuka, Gabriel Vichera

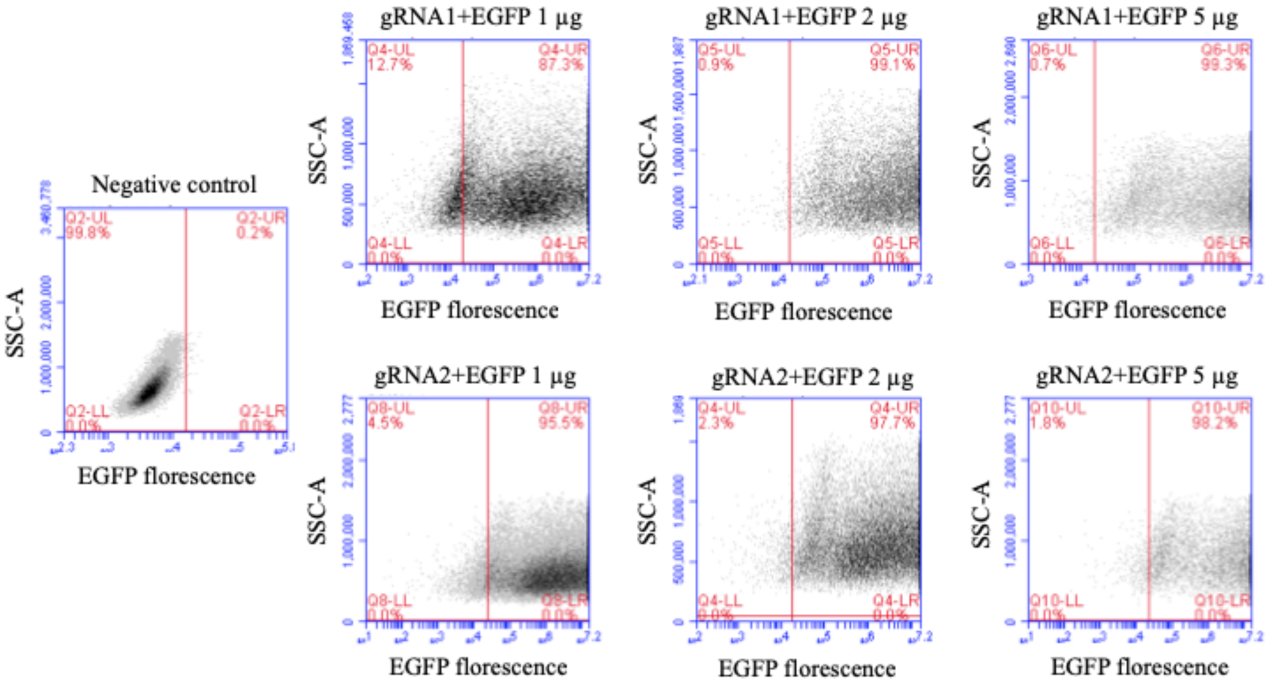

Supplementary Figure S1. Flow cytometry analysis of EGFP expression in horse fetal fibroblasts after nucleofection. The negative control without nucleofection was used to determine the positive quadrant to the right of the red strip. % of EGFP positive cells is shown on the top right in each condition. SSC-A, Side Scatter.

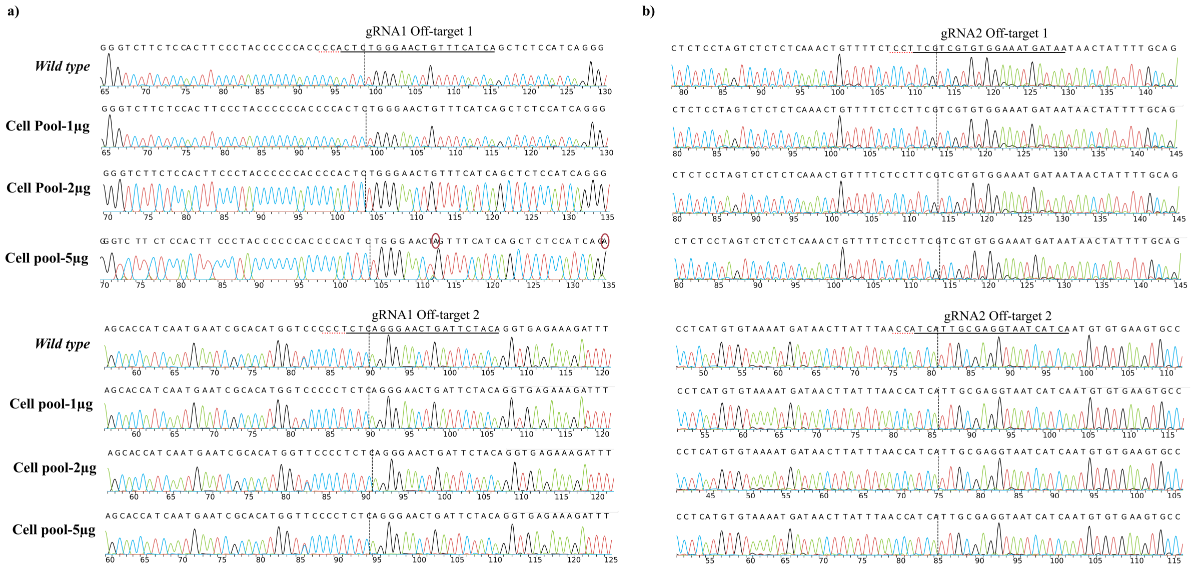

Supplementary Figure S2. Off-target analysis in cell pools. a) Histograms with nucleotide sequence data of two gRNA1 putative off-targets. The red circles show two A insertions in G1-5µg group. No other editions were detected. b) Histograms with nucleotide sequence data of two gRNA2 putative off-targets. No editions were detected in any condition.

a)

<table>
<tr><th>gRNA</th><th>DNA</th><th>Cell Clone</th><th>Allele</th><th>Sequence (Coding Strand)</th><th>Mutation Type</th><th>Genotype Mutation</th><th>MSTN a.a ..73-104..</th></tr>
<tr><td></td><td></td><td>wt</td><td>-</td><td>CAACTTTTGCCCAAAGCTCCTCCACTC|CGGGAACTGATTGATCAG</td><td>wt</td><td>wt</td><td>..SKDAIRQLLPKAPPLRELIDQYDVQRDDSSDG..</td></tr>
<tr><td rowspan="52">gRNA 1</td><td rowspan="14">1 µg</td><td rowspan="2">C01</td><td>#1</td><td>CAACTTTTGCCCAAAGCTCCTCCACTC|CGGGAACTGATTGATCAG</td><td>wt</td><td>wt</td><td>..SKDAIRQLLPKAPPLRELIDQYDVQRDDSSDG..</td></tr>
<tr><td>#2</td><td>CAACTTTTGCCCAAAGCTCCTCCACT-|CGGGAACTGATTGATCAG</td><td>Del-1</td><td>DelC</td><td>..SKDAIRQLLPKAPPLGN*</td></tr>
<tr><td rowspan="2">C02</td><td>#1</td><td>CAACTTTTGCCCAAAGCTCCTCCACTC|CGCGGGAACTGATTGATCAG</td><td>Ins+2</td><td>InsCG</td><td>..SKDAIRQLLPKAPPLAGN*</td></tr>
<tr><td>#2</td><td>CAACTTTTGCCCAAAGCTCCTCCACTC|CGCGGGAACTGATTGATCAG</td><td>Ins+2</td><td>InsCG</td><td>..SKDAIRQLLPKAPPLAGN*</td></tr>
<tr><td rowspan="2">C05</td><td>#1</td><td>CAACTTTTGCCCAAAGCTCCTCCACTC|CGGGAACTGATTGATCAG</td><td>wt</td><td>wt</td><td>..SKDAIRQLLPKAPPLRELIDQYDVQRDDSSDG..</td></tr>
<tr><td>#2</td><td>CAACTTTTGCCCAAAGCTCCTCCACTC|CGGGAACTGATTGATCAG</td><td>wt</td><td>wt</td><td>..SKDAIRQLLPKAPPLRELIDQYDVQRDDSSDG..</td></tr>
<tr><td rowspan="2">C08</td><td>#1</td><td>CAACTTTTGCCCAAAGCTCCTCCACTC|CGGGAACTGATTGATCAG</td><td>wt</td><td>wt</td><td>..SKDAIRQLLPKAPPLRELIDQYDVQRDDSSDG..</td></tr>
<tr><td>#2</td><td>CAACTTTTGCCCAAAGCTCCTCCACTC|CGGGAACTGATTGATCAG</td><td>wt</td><td>wt</td><td>..SKDAIRQLLPKAPPLRELIDQYDVQRDDSSDG..</td></tr>
<tr><td rowspan="2">C13</td><td>#1</td><td>CAACTTTTGCCCAAAGCTCCTCCACTC|CGGGAACTGATTGATCAG</td><td>wt</td><td>wt</td><td>..SKDAIRQLLPKAPPLRELIDQYDVQRDDSSDG..</td></tr>
<tr><td>#2</td><td>CAACTTTTGCCCAAAGCTCCTCCACTC|CGGGAACTGATTGATCAG</td><td>wt</td><td>wt</td><td>..SKDAIRQLLPKAPPLRELIDQYDVQRDDSSDG..</td></tr>
<tr><td rowspan="2">C17</td><td>#1</td><td>CAACTTTTGCCCAAAGCTCCTCCACTC|CGGGAACTGATTGATCAG</td><td>wt</td><td>wt</td><td>..SKDAIRQLLPKAPPLRELIDQYDVQRDDSSDG..</td></tr>
<tr><td>#2</td><td>CAACTTTTGCCCAAAGCTCCTCCACTC|CGGGAACTGATTGATCAG</td><td>wt</td><td>wt</td><td>..SKDAIRQLLPKAPPLRELIDQYDVQRDDSSDG..</td></tr>
<tr><td rowspan="2">C23</td><td>#1</td><td>CAACTTTTGCCCAAAGCTCCTCCACTC|CGCGGGAACTGATTGATCAG</td><td>Ins+2</td><td>InsCG</td><td>..SKDAIRQLLPKAPPLAGN*</td></tr>
<tr><td>#2</td><td>CAACTTTTGCCCAAAGCTCCTCCACTC|CGCGGGAACTGATTGATCAG</td><td>Ins+2</td><td>InsCG</td><td>..SKDAIRQLLPKAPPLAGN*</td></tr>
<tr><td rowspan="14">2 µg</td><td rowspan="2">C03</td><td>#1</td><td>CAACTTTTGCCCAAAGCTCCTCCACTC|CGCGGGAACTGATTGATCAG</td><td>Ins+2</td><td>InsCG</td><td>..SKDAIRQLLPKAPPLAGN*</td></tr>
<tr><td>#2</td><td>CAACTTTTGCCCAAAGCTCCTCCACTC|CGCGGGAACTGATTGATCAG</td><td>Ins+2</td><td>InsCG</td><td>..SKDAIRQLLPKAPPLAGN*</td></tr>
<tr><td rowspan="2">C06</td><td>#1</td><td>CAACTTTTGCCCAAAGCTCCTCCACTC|+57-GGGAACTGATTGATCAG</td><td>Del-1/Ins+57</td><td>delC-InsCGCTTTGATCTTTT…</td><td>..SKDAIRQLLPKAPPLRFDLFYGV*</td></tr>
<tr><td>#2</td><td>CAACTTTTGCCCAAAGCTCCTCCACTC|------------------</td><td>Del-33</td><td>delCGGGAACTGATTGATCA…</td><td>..SKDAIRQLLPKAPPL-----------DDSSDG..</td></tr>
<tr><td rowspan="2">C10</td><td>#1</td><td>CAACTTTTGCCCAAAGCTCCTCCACTC|CGGGAACTGATTGATCAG</td><td>wt</td><td>wt</td><td>..SKDAIRQLLPKAPPLRELIDQYDVQRDDSSDG..</td></tr>
<tr><td>#2</td><td>CAACTTTTGCCCAAAGCTCCTCCACTC|CGGGAACTGATTGATCAG</td><td>wt</td><td>wt</td><td>..SKDAIRQLLPKAPPLRELIDQYDVQRDDSSDG..</td></tr>
<tr><td rowspan="2">C11</td><td>#1</td><td>CAACTTTTGCCCAAAGCTCCTCCACTC|CGGGAACTGATTGATCAG</td><td>wt</td><td>wt</td><td>..SKDAIRQLLPKAPPLRELIDQYDVQRDDSSDG..</td></tr>
<tr><td>#2</td><td>CAACTTTTGCCCAAAGCTCCTCCACTC|CGGGAACTGATTGATCAG</td><td>wt</td><td>wt</td><td>..SKDAIRQLLPKAPPLRELIDQYDVQRDDSSDG..</td></tr>
<tr><td rowspan="2">C13</td><td>#1</td><td>CAACTTTTGCCCAAAGCTCCTCCACT-|CGGGAACTGATTGATCAG</td><td>Del-1</td><td>DelC</td><td>..SKDAIRQLLPKAPPLGN*</td></tr>
<tr><td>#2</td><td>CAACTTTTGCCCAAAGCTCCTCCACT-|CGGGAACTGATTGATCAG</td><td>Del-1</td><td>DelC</td><td>..SKDAIRQLLPKAPPLGN*</td></tr>
<tr><td rowspan="2">C15</td><td>#1</td><td>CAACTTTTGCCCAAAGCTCCTCCACTC|GCCGGGAACTGATTGATCAG</td><td>Ins+2</td><td>InsGC</td><td>..SKDAIRQLLPKAPPLRGN*</td></tr>
<tr><td>#2</td><td>CAACTTTTGCCCAAAGCTCCTCCACTC|AGCGGGAACTGATTGATCAG</td><td>Ins+2</td><td>InsAG</td><td>..SKDAIRQLLPKAPPLSGN*</td></tr>
<tr><td rowspan="2">C27</td><td>#1</td><td>CAACTTTTGCCCAAAGCTCCTCCACT-|CGGGAACTGATTGATCAG</td><td>Del-1</td><td>DelC</td><td>..SKDAIRQLLPKAPPLGN*</td></tr>
<tr><td>#2</td><td>CAACTTTTGCCCAAAGCTCCTCCACT-|TCGGGAACTGATTGATCAG</td><td>Del-1/Ins+1</td><td>DelC/InsT</td><td>..SKDAIRQLLPKAPPLWELIDQYDVQRDDSSDG..</td></tr>
<tr><td rowspan="16">5 µg</td><td rowspan="2">C01</td><td>#1</td><td>CAACTTTTGCCCAAAGCTCCTCCACT-|CGGGAACTGATTGATCAG</td><td>Del-1</td><td>DelC</td><td>..SKDAIRQLLPKAPPLGN*</td></tr>
<tr><td>#2</td><td>CAACTTTTGCCCAAAGCTCCTCCACTC|-------------ATCAG</td><td>Del-13</td><td>DelCGGGAACTGATTG</td><td>..SKDAIRQLLPKAPPLISTMSREMTAVMALWKM..</td></tr>
<tr><td rowspan="2">C02</td><td>#1</td><td>CAACTTTTGCCCAAAGCTCCTCCACTC|+57CGGGAACTGATTGATCAG</td><td>Ins+68</td><td>InsGTTGGGTGCACGAGTGGG…</td><td>..SKDAIRQLLPKAPPLVGCTSGLHRTGSQQR*</td></tr>
<tr><td>#2</td><td>CAACTTTTGCCCAAAGCTCCTCCACTC|+57CGGGAACTGATTGATCAG</td><td>Ins+68</td><td>InsGTTGGGTGCACGAGTGGG…</td><td>..SKDAIRQLLPKAPPLVGCTSGLHRTGSQQR*</td></tr>
<tr><td rowspan="2">C07</td><td>#1</td><td>CAACTTTTGCCCAAAGCTCCTCCACTC|CGGGAACTGATTGATCAG</td><td>wt</td><td>wt</td><td>..SKDAIRQLLPKAPPLRELIDQYDVQRDDSSDG..</td></tr>
<tr><td>#2</td><td>CAACTTTTGCCCAAAGCTCCTCCACTC|CGGGAACTGATTGATCAG</td><td>wt</td><td>wt</td><td>..SKDAIRQLLPKAPPLRELIDQYDVQRDDSSDG..</td></tr>
<tr><td rowspan="2">C08a</td><td>#1</td><td>----------------------------|-------------ATCAG</td><td>del-37/+13</td><td>delTGCTATTAGACAACTTTTG…</td><td>..SKESVRCPER*</td></tr>
<tr><td>#2</td><td>CAACTTTTGCCCAAAGCTCCTCCACTC|--GGAACTGATTGATCAG</td><td>Del-2</td><td>DelGC</td><td>..SKDAIRQLLPKAPPLGTD*</td></tr>
<tr><td rowspan="2">C08b</td><td>#1</td><td>CAACTTTTGCCCAAAGCTCCTCCACT-|CGGGAACTGATTGATCAG</td><td>Del-1</td><td>DelC</td><td>..SKDAIRQLLPKAPPLGN*</td></tr>
<tr><td>#2</td><td>----------------------------|------------------</td><td>Del-32/+20</td><td>DelTAGACAACTTTTGCCCAAA…</td><td>..SKDAI-----------------DVQRDDSSDG..</td></tr>
<tr><td rowspan="2">C09</td><td>#1</td><td>CAACTTTTGCCCAAAGCTCCTCCACT-|CGGGAACTGATTGATCAG</td><td>Del-1</td><td>DelC</td><td>..SKDAIRQLLPKAPPLGN*</td></tr>
<tr><td>#2</td><td>CAACTTTTGCCCAAAGCTCCTCCACT-|CGGGAACTGATTGATCAG</td><td>Del-1</td><td>DelC</td><td>..SKDAIRQLLPKAPPLGN*</td></tr>
<tr><td rowspan="2">C12</td><td>#1</td><td>CAACTTTTGCCCAAAGCTCCTCCACTC|CGGGAACTGATTGATCAG</td><td>wt</td><td>wt</td><td>..SKDAIRQLLPKAPPLRELIDQYDVQRDDSSDG..</td></tr>
<tr><td>#2</td><td>CAACTTTTGCCCAAAGCTCCTCCACTC|CGGGAACTGATTGATCAG</td><td>wt</td><td>wt</td><td>..SKDAIRQLLPKAPPLRELIDQYDVQRDDSSDG..</td></tr>
<tr><td rowspan="2">C14</td><td>#1</td><td>CAACTTTTGCCCAAAGCTCCTCCACT-|CGGGAACTGATTGATCAG</td><td>Del-1</td><td>DelC</td><td>..SKDAIRQLLPKAPPLGN*</td></tr>
<tr><td>#2</td><td>CAACTTTTGCCCAAAGCTCCTCCACT-|CGGGAACTGATTGATCAG</td><td>Del-1</td><td>DelC</td><td>..SKDAIRQLLPKAPPLGN*</td></tr>
</table>

b)

gRNA	DNA	Cell Clone	Allele	Sequence	Mutation Type	Genotype Mutation	MSTN a.a ..100-130..
		wt		TGATGATTACCACGCGA|CGACGGAAACAATCATTACCATGCCTA	*wt*	*wt*	..DSSDGSLEDDDYHATTETIITMPTESDLLMQ..
gRNA2	1 µg	C02	#1	TGATGATTACCACGCGA|CGACGGAAACAATCATTACCATGCCTA	*wt*	*wt*	..DSSDGSLEDDDYHATTETIITMPTESDLLMQ..
			#2	TGATGATTACCAC----|CGACGGAAACAATCATTACCATGCCTA	del-4	delGCGA	..DSSDGSLEDDDYH**RRKQSLPCLQSLIF***
		C08	#1	TGATGATTACCACGCGA|CCGACGGAAACAATCATTACCATGCCTA	ins+1	insC	..DSSDGSLEDDDYHAT**DGNNHYHAYRV***
			#2	TGATGATTACCACGCG-|CGACGGAAACAATCATTACCATGCCTA	del-1	delA	..DSSDGSLEDDDYHA**RRKQSLPCLQSLIF***
		C12	#1	TGATGATTACCACGCGA|GCGACGGAAACAATCATTACCATGCCTA	ins+1	insG	..DSSDGSLEDDDYHA**SDGNNHYHAYRV***
			#2	TGATGATTACCAC----|CGACGGAAACAATCATTACCATGCCTA	del-4	delGCGA	..DSSDGSLEDDDYH**RRKQSLPCLQSLIF***
		C13	#1	TGATGATTACCACGCGA|CGACGGAAACAATCATTACCATGCCTA	*wt*	*wt*	..DSSDGSLEDDDYHATTETIITMPTESDLLMQ..
			#2	TGATGATTACCACGCGA|CGACGGAAACAATCATTACCATGCCTA	*wt*	*wt*	..DSSDGSLEDDDYHATTETIITMPTESDLLMQ..
		C14	#1	TGATGATTACCACGCGA|---CGGAAACAATCATTACCATGCCTA	del-3	delCGA	..DSSDGSLEDDDYHA-TETIITMPTESDLLMQ..
			#2	TGATGATTACCACGCGA|---CGGAAACAATCATTACCATGCCTA	del-3	delCGA	..DSSDGSLEDDDYHA-TETIITMPTESDLLMQ..
		C19	#1	TGATGATTACCACGCGA|CCGACGGAAACAATCATTACCATGCCTA	ins+1	insC	..DSSDGSLEDDDYHAT**DGNNHYHAYRV***
			#2	TGATGATTACCACGCG-|CGACGGAAACAATCATTACCATGCCTA	del-1	delA	..DSSDGSLEDDDYHA**RRKQSLPCLQSLIF***
	2 µg	C02	#1	TGATGATTACCACGCGA|CCGACGGAAACAATCATTACCATGCCTA	ins+1	insC	..DSSDGSLEDDDYHAT**DGNNHYHAYRV***
			#2	TGATGATTACCACGCGA|CCGACGGAAACAATCATTACCATGCCTA	ins+1	insC	..DSSDGSLEDDDYHAT**DGNNHYHAYRV***
		C03	#1	TGATGATTACCACGCG-|CGACGGAAACAATCATTACCATGCCTA	del-1	delA	..DSSDGSLEDDDYHA**RRKQSLPCLQSLIF***
			#2	TGATGATTACCACGCG-|CGACGGAAACAATCATTACCATGCCTA	del-1	delA	..DSSDGSLEDDDYHA**RRKQSLPCLQSLIF***
		C08	#1	TGATGATTACCACGCGA|---CGGAAACAATCATTACCATGCCTA	del-3	delCGA	..DSSDGSLEDDDYHA-TETIITMPTESDLLMQ..
			#2	TGATGATTACCACGCGA|ACGACGGAAACAATCATTACCATGCCTA	ins+1	insA	..DSSDGSLEDDDYHA**NDGNNHYHAYRV***
		C19	#1	TGATGATTACCACGCGA|---CGGAAACAATCATTACCATGCCTA	del-3	delCGA	..DSSDGSLEDDDYHA-TETIITMPTESDLLMQ..
			#2	TGATGATTACCACGCGA|ACGACGGAAACAATCATTACCATGCCTA	ins+1	insA	..DSSDGSLEDDDYHA**NDGNNHYHAYRV***
		C23	#1	TGATGATTACCACGCG-|CGACGGAAACAATCATTACCATGCCTA	del-1	delA	..DSSDGSLEDDDYHA**RRKQSLPCLQSLIF***
			#2	TGATGATTACCACGCGA|GCGACGGAAACAATCATTACCATGCCTA	ins+1	insG	..DSSDGSLEDDDYHA**SDGNNHYHAYRV***
		C28	#1	TGATGATTACCACGC--|CGACGGAAACAATCATTACCATGCCTA	Del-2	DelGA	..DSSDGSLEDDDYHA**DGNNHYHAYRV***
			#2	TGATGATTACCACGCGA|+69CGACGGAAACAATCATTACCATGCCTA	Ins+69	InsACTAGACCATGTTT…	..DSSDGSLEDDDYHAT**TLDHV***
		C29	#1	TGATGATTACCACGCGA|+59CGACGGAAACAATCATTACCATGCCTA	ins+59	insGAAGGAATGAAGA…	..DSSDGSLEDDDYHA**RRNEEGGHMMHC***
			#2	TGATGATTACCACGCGA|+59CGACGGAAACAATCATTACCATGCCTA	ins+59	insGAAGGAATGAAGA…	..DSSDGSLEDDDYHA**RRNEEGGHMMHC***
	5 µg	C02	#1	TGATGATTACC------|CGACGGAAACAATCATTACCATGCCTA	del-6	delACGCGA	..DSSDGSLEDDDY--**P**TETIITMPTESDLLMQ..
			#2	TGATGATTACC------|CGACGGAAACAATCATTACCATGCCTA	del-6	delACGCGA	..DSSDGSLEDDDY--**P**TETIITMPTESDLLMQ..
		C03	#1	TGATGATTACCACGCGA|CCGACGGAAACAATCATTACCATGCCTA	ins+1	insC	..DSSDGSLEDDDYHAT**DGNNHYHAYRV***
			#2	TGATGATTACCACGCGA|CCGACGGAAACAATCATTACCATGCCTA	ins+1	insC	..DSSDGSLEDDDYHAT**DGNNHYHAYRV***
		C05	#1	TGATGATTACCACGCGA|+111CGACGGAAACAATCATTACCATGCCTA	ins+111	insACCGAAACGCGCG..	..DSSDGSLEDDDYHAT**PKRARRRGLAIRLFL***
			#2	TGATGATTACCACGCGA|+184(-73)TGTTGTTCTAGTGTTTATGAGAA	ins+184/del-73	insAACGGACATCGTG..	..DSSDGSLEDDDYHAT**DGNNHYHAYRV***
		C07	#1	-----------------|----------------------------	del-19/+17	delGATGATGATTACCA…	..DSSDGSLE**TMPTECK***
			#2	-----------------|----------------------------	del-19/+17	delGATGATGATTACCA…	..DSSDGSLE**TMPTECK***
		C10	#1	TGATGATTACCACGCGA|ACGACGGAAACAATCATTACCATGCCTA	ins+1	insA	..DSSDGSLEDDDYHA**NDGNNHYHAYRV***
			#2	TGATGATTACCACGC--|CGACGGAAACAATCATTACCATGCCTA	del-2	delGA	..DSSDGSLEDDDYHA**DGNNHYHAYRV***
		C12	#1	TGATGATTACCACGCGA|GCGACGGAAACAATCATTACCATGCCTA	ins+1	insG	..DSSDGSLEDDDYHA**SDGNNHYHAYRV***
			#2	TGATGATTACCACGCGA|GCGACGGAAACAATCATTACCATGCCTA	ins+1	insG	..DSSDGSLEDDDYHA**SDGNNHYHAYRV***
		C13	#1	TGATGATTACCACGCGA|ACGACGGAAACAATCATTACCATGCCTA	ins+1	insA	..DSSDGSLEDDDYHA**NDGNNHYHAYRV***
			#2	TGATGATTACCACGCGA|-----GAAACAATCATTACCATGCCTA	del-5	delCGACG	..DSSDGSLEDDDYHA**RNNHYHAYRV***
		C14	#1	TGATGATTACCACGCGA|CCGACGGAAACAATCATTACCATGCCTA	ins+1	insC	..DSSDGSLEDDDYHAT**DGNNHYHAYRV***
			#2	TGATGATTACCACGCGA|CCGACGGAAACAATCATTACCATGCCTA	ins+1	insC	..DSSDGSLEDDDYHAT**DGNNHYHAYRV***
		C16	#1	TGATGATTACCACGC--|CGACGGAAACAATCATTACCATGCCTA	del-2	delGA	..DSSDGSLEDDDYHA**DGNNHYHAYRV***
			#2	TGATGATTACCACGC--|CGACGGAAACAATCATTACCATGCCTA	del-2	delGA	..DSSDGSLEDDDYHA**DGNNHYHAYRV***
		C18	#1	TGATGATTACCACGCGA|+62CGACGGAAACAATCATTACCATGCCTA	ins+62	insTCGACCAAAATCCCT…	..DSSDGSLEDDDYHA**IDQNPLT***
			#2	TGATGATTACCACGCGA|+62CGACGGAAACAATCATTACCATGCCTA	ins+62	insTCGACCAAAATCCCT…	..DSSDGSLEDDDYHA**IDQNPLT***
		C21	#1	TGATGATTACCACGCGA|CCGACGGAAACAATCATTACCATGCCTA	ins+1	insC	..DSSDGSLEDDDYHAT**DGNNHYHAYRV***
			#2	TGATGATTACCACGCGA|TCGACGGAAACAATCATTACCATGCCTA	ins+1	insT	..DSSDGSLEDDDYHA**IDGNNHYHAYRV***
		C22	#1	TGATGATTACCACGCGA|-GACGGAAACAATCATTACCATGCCTA	del-1	delC	..DSSDGSLEDDDYHA**RRKQSLPCLQSLIF***
			#2	TGATGATTACCACGCGA|-GACGGAAACAATCATTACCATGCCTA	del-1	delC	..DSSDGSLEDDDYHA**RRKQSLPCLQSLIF***
		C23	#1	TGATGATTACCACGCGA|CCGACGGAAACAATCATTACCATGCCTA	ins+1	insC	..DSSDGSLEDDDYHAT**DGNNHYHAYRV***
			#2	TGATGATTACCACGCGA|CCGACGGAAACAATCATTACCATGCCTA	ins+1	insC	..DSSDGSLEDDDYHAT**DGNNHYHAYRV***

Supplementary Table S1. Isolated cell clones' sequences of gRNA target regions in exon 1 of *MSTN* gene. DNA sequences were analyzed with Synthego or Indigo the in case of InDels of more than 50 bases. a) Nucleotide and aminoacid sequences of gRNA1 target region of selected cell clones. b) Nucleotide and aminoacid sequences of gRNA2 target region of selected cell clones. Ins, insertions; del, deletions. gRNA1 and gRNA2 sequences are shown in blue with the vertical line pointing the cut site of Cas9 for each gRNA. The protospacer-adjacent motif (PAM) is labeled in orange. The nucleotide insertions are shown in pink.

* correspond to stop codon appearance in the MSTN protein sequence.
